# Robotic cardiac surgery for removal of iliac venous stent embolized in the right cardiac chambers: a case report

**DOI:** 10.31744/einstein_journal/2024RC0943

**Published:** 2024-11-06

**Authors:** Robinson Poffo, Andressa Cristina Sposato Louzada, Sergio Augusto Fudaba Curcio, Marcelo Passos Teivelis, Nelson Wolosker

**Affiliations:** 1 Hospital Israelita Albert Einstein São Paulo SP Brazil Hospital Israelita Albert Einstein, São Paulo, SP, Brazil.; 2 Hospital Israelita Albert Einstein Faculdade Israelita de Ciências da Saúde Albert Einstein São Paulo SP Brazil Faculdade Israelita de Ciências da Saúde Albert Einstein, Hospital Israelita Albert Einstein, São Paulo, SP, Brazil.; 3 Universidade de São Paulo Faculdade de Medicina São Paulo SP Brazil Faculdade de Medicina, Universidade de São Paulo, São Paulo, SP, Brazil.

**Keywords:** Robotic surgical procedures, Minimally invasive surgical procedures, Endovascular procedures, Angioplasty, Dyspnea, Stents/adverse effects

## Abstract

A 42-year-old female patient with a surgical history of iliac venous angioplasty with stenting developed dyspnea on exertion 9 months later. Chest computed tomography angiography revealed a fractured vascular stent in the right cardiac chamber. Doppler echocardiography confirmed that the stent was anchored by the tricuspid valve, causing mild obstruction of the right ventricular filling. The patient underwent robot-assisted cardiac surgery with stent removal, annuloplasty under general anesthesia, and cardiopulmonary bypass via an axillary incision. No sternotomy, cardioplegia, or aortic clamping was required. The right atrium was opened, and no surgical or anesthetic complications occurred. The patient was extubated in the operating room, with no requirement for vasoactive drugs after surgery. She was discharged on the fifth postoperative day in a good general condition, eupneic, and without lower-limb venous symptoms. Re-do iliac venous angioplasty was not necessary.

## INTRODUCTION

Stent embolization in the heart after iliac venous angioplasty to treat May-Thurner syndrome is a rare but severe complication, with only 12 such reports available in literature. Among these, only one case of endovascular treatment was successful.^(1)^ Endovascular attempts failed in four patients who underwent open-heart surgery with cardiopulmonary bypass (CPB).^(2-4)^ The remaining seven patients were directly treated by open-heart surgery, allowing the concomitant repair of intracardiac structural damage, but with high morbidity and mortality.^(4-8)^

We report a case of venous stent embolization in the right cardiac chamber that was successfully treated by minimally invasive robotic cardiac surgery, which waived the need for sternotomy, cardioplegia, and aortic clamping. To the best of our knowledge, this is the first report on the successful implementation of this treatment strategy.

## CASE REPORT

A 42-year-old female patient with a previous history of iliac venous angioplasty with stenting (Zilver^®^ Vena stent 14×100mm, Cook Medical, Indiana, United States) to treat May-Thurner syndrome presented with dyspnea on exertion 9 months later. The patient was in a good general condition, hemodynamically stable, and eupneic on room air. Cardiac and pulmonary auscultation findings were normal.

**Figure 1:**
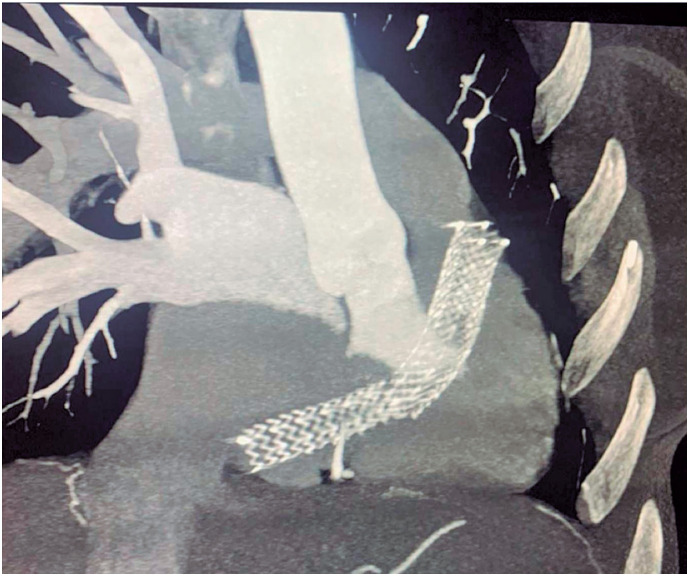
Chest CT angiogram showing the vascular stent in the right heart chambers

During initial investigations, chest radiography revealed an intracardiac foreign body. Further investigations using chest computed tomography (CT) angiography (Figure 1), showing a fractured stent with its proximal edge in the right atrium (RA), near the junction with the inferior vena cava, and its distal edge in the right ventricle (RV), 0.8cm from the pulmonary valve. Due to the potential risk of valve injury, a Doppler echocardiogram was performed, which showed slight dilation of the right cardiac chambers. It also confirmed the location of the stent between the RA and RV, anchored by the tricuspid valve (TV), causing slight obstruction of the right ventricular filling. The global systolic function was preserved, and the pulmonary arterial systolic pressure was 19mmHg.

**Figure 2:**
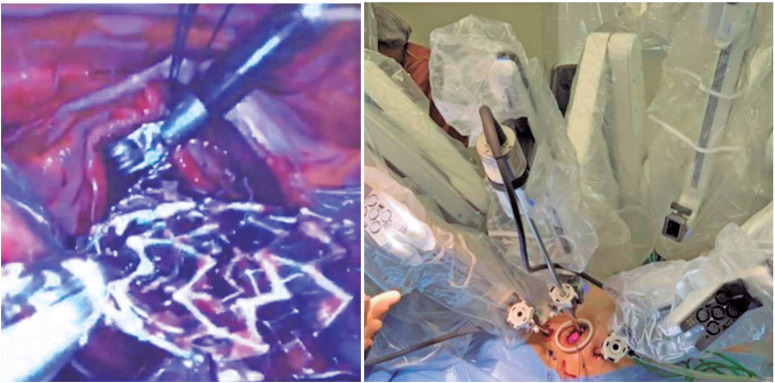
On the left, Zilver^®^ Vena stent being removed from the right cardiac chambers. On the right, the docked daVinci robotic system

The patient underwent robotic cardiac surgery on account of the valve injury, with no attempts at endovascular treatment. The robotic technique has been described elsewhere.^(9)^ Briefly, the right internal jugular vein, femoral vein, and femoral artery (RFA) were cannulated percutaneously under general anesthesia and CPB. A 3.5cm incision was made from the anterior to mid-axillary line, at the fourth intercostal space, where robotic optics were inserted, and two trocars for robotic arms were placed (da Vinci^®^ Surgical System, Intuitive Surgical, California, United States) (Figure 2). After isolation of the superior and inferior vena cava, an incision was made into the lateral wall of the RA. Cardioplegic solution or aortic clamp were not used since the left chambers were preserved. The stent was identified and removed (Figure 2), and a cleft in the anterior leaflet of the TV was diagnosed and closed with a running suture of 5.0 Gore-Tex^®^. The RA was subsequently closed and CPB was discontinued. Transesophageal echocardiography (TEE) showed moderate TV insufficiency; thus, after restarting CPB, the RA was reopened and tricuspid annuloplasty was performed using the DeVega technique, with a satisfactory saline test. The final TEE showed a competent TV. The total CPB duration was 180 min, and the heart maintained a sinus rhythm throughout the procedure. Decannulation was performed, and the RFA was closed using the Perclose ProGlide™ Suture-Mediated Closure System (Abbott). The chest was then drained and sutured in plane. The patient received 4000mL of PlasmaLyte (Baxter international), 200mL of albumin, 4g of fibrinogen, and 1 platelet apheresis guided by thromboelastogram. Additionally, 1107mL of total blood were infused after recovery using the auto-transfusion device (Cell Saver^®^, Elite+), and heparin was reversed with protamine. The surgery lasted 6 hours 20 min, and anesthesia lasted 9 hours 10 min.

No complications were evident, and the patient was extubated in the operating room, with no requirement for vasoactive drugs after surgery.

The patient was stable and was transferred to the intensive care unit (ICU). On the first postoperative day (POD), the patient was transferred to a semi-ICU. On the third POD, the chest drain was removed, chemical prophylaxis for venous thromboembolism was initiated, and the patient was transferred to a regular hospital bed. On the fourth POD, Doppler echocardiography revealed normal-sized right cardiac chambers, TV with signs of plasty, satisfactory opening, and mild reflux (7mmHg and 3mmHg for maximum and mean diastolic gradients). Venous Doppler ultrasound showed the inferior vena cava, iliac veins, and deep veins of the lower limbs with preserved caliber, walls, and compressibility, without thrombi or signs of incompetence. The intersection of the right common iliac artery with the left common iliac vein showed a preserved spectrum and velocity, before and after, which negated the requirement for a re-do venous angioplasty. On the fifth POD, the patient was discharged in good general condition, eupneic, and without lower-limb venous symptoms.

This study was approved by the Ethics Committee of the *Hospital Israelita Albert Einstein* (CAAE: 56235122.4.0000.0071; #5.288.106) on March 12, 2022. The patient was invited to participate in the study and signed a consent form for publication of this case report and the use of images.

## DISCUSSION

We present the case of an adult female patient with dyspnea on exertion, whose only positive medical history was iliac venous stenting.

Diagnosing stent embolization in the heart as the etiology of dyspnea can be challenging and requires high suspicion. The investigation was initiated with chest radiography and was complemented by CT angiography, which detailed the intracardiac foreign body. Additional Doppler echocardiography allowed valve and myocardial function evaluation, thus confirming the etiology of the dyspnea, and guiding the development of the best treatment strategy. Therapeutic decisions must thus be made by a multidisciplinary team of emergency physicians, cardiologists, and cardiac and vascular surgeons.

Despite one report of the successful endovascular removal of an intracardiac foreign body,^(1)^ most attempts have been unsuccessful.^(2-4,7)^ Further, this approach prevents simultaneous treatment of structural injuries. We decided to proceed directly with cardiac surgery on account of the TV damage.

As open cardiac surgery is associated with severe postoperative complications, including, stroke, atrial fibrillation, cardiac tamponade, and sternotomy-related complications,^(4,7)^ our multidisciplinary team chose the effective, less invasive, and safer robotic approach.^(10)^

The uneventful postoperative course, and hospital discharge on the fifth POD in good general condition highlights the safety and benefits of robotic cardiac surgery. Notably, reassessment of the patient negated the requirement of another venous angioplasty procedure.

Although stent migration is a rare complication, it is a serious life threatening outcome. We recommend that robotic cardiac surgery should be considered the first option for removing foreign bodies associated with intracardiac injuries. Further, additional research on the standardization of the site and size of venous stent placement and imaging follow-up, as well as implementing best-practice guidelines is essential for best clinical outcomes.

## CONCLUSION

Embolization of venous stents in the heart represents a challenging diagnosis of dyspnea, and its management should involve a multidisciplinary team. Robotic cardiac surgery is a minimally invasive and less morbid procedure for the removal of foreign bodies associated with structural intracardiac injuries.
